# Commissioning and Assessment of Radiation Field and Dose Inhomogeneity for a Dual X-ray Tube Cabinet Irradiator: To Ensure Accurate Dosimetry in Radiation Biology Experiments

**DOI:** 10.1016/j.adro.2024.101486

**Published:** 2024-03-07

**Authors:** Mitchell Polizzi, Kristoffer Valerie, Siyong Kim

**Affiliations:** a Department of Radiation Oncology, Virginia Commonwealth University, Richmond, Virginia; b Massey Cancer Center, Virginia Commonwealth University, Richmond, Virginia

## Abstract

**Purpose:**

Standardization of x-ray cabinet irradiator dose, geometry, and calibration reporting is an ongoing process. Multi-tube designs have been introduced into the preclinical market and give a theoretical benefit but have not been widely assessed for use in preclinical irradiation conditions. The aim of this study was to report our experience commissioning a dual x-ray source cabinet irradiator (CIXD, Xstrahl Limited, United Kingdom) and assess the dose distribution for various experimental conditions.

**Methods and Materials:**

Half-value layer (HVL) measurement, profile measurements, and output calibration were performed using a calibrated ion chamber. Constancy measurements were performed twice daily over 2 weeks to assess output fluctuations. Film measurements were completed using solid water to assess percent depth dose and homogeneity within the field and within variable thicknesses of solid water and phosphate-buffered saline solution. Film measurements were repeated for various arrangements of petri dishes filled with phosphate-buffered saline or water and in a 3D-printed mouse phantom.

**Results:**

The x-ray tubes had a measured in-air output of 1.27 Gy/min. The HVL was 1.7 mm Cu. The upper and lower tubes both exhibited the heel effect, but when operated simultaneously, the effect was reduced. Ion chamber measurements revealed a 15% dose inhomogeneity within the tray area (18 × 18 cm^2^). Film measurements in the petri dishes indicated minor nonuniformities in the arrangements of the experimental apparatus. Measurements from the mouse phantom with film agreed with ion chamber measurements for various phantom placements and orientations.

**Conclusions:**

X-ray cell culture and animal irradiation with dual tube cabinet irradiation is efficient and robust when using established dosimetric tools to confirm output and homogeneity. The conditions assumed for calibrations are often not maintained during experiments. We have confirmed that inhomogeneities are present for single-tube use; however, they are reduced with simultaneous tube use. Additional dosimetric monitoring should be performed for each unique irradiation setup.

## Introduction

Radiation biology has provided innumerable findings and conclusions that have transformed radiation oncology throughout the years.^1^ The body of research in radiation biology has expanded our understanding of the underlying mechanisms of tumor control^2^ and normal tissue complications^3^ and has led to the adoption of new techniques and therapeutics.^4^^,^^5^ Historically, cell irradiation has been completed using cesium-137 or cobalt-60, which have included well-known dose rates and beam uniformity.^6^, ^7^, ^8^ Various studies have found general agreement between manufacturer-provided data and their own measurements;^9^^,^^10^ however, as noted in a meta-analysis, the majority of publications have lacked reporting of physics and dosimetry parameters.^11^ This has led to a lack of reproducibility in studies and a concern that findings cannot be pooled for further analysis. In addition, the well-characterized Cs-137 animal irradiators are being replaced, owing to increasing concerns and the costs of radioactive source security.^12^^,^^13^ The lack of reproducibility in radiobiological experiments could potentially be exacerbated by the introduction of x-ray sources, with several groups reporting their dosimetry work for single x-ray tube equipment, which can be highly variable owing to the x-ray energy spectrum, dose rate, and other dosimetric variables.^14^, ^15^, ^16^, ^17^, ^18^

X-ray sources provide a suitable replacement for Cs-137 sources, with benefits that include generating x-rays through thermionic emission and bremsstrahlung only when needed, instead of constant radioactive decay. The cabinets are often self-shielding, like Cs-137 sources, but owing to a lower x-ray spectrum, the shielding requirement is lower. There is no need for additional security or monitoring. However, there is a prominent downside to the x-ray source. Due to the design of modern x-ray tubes, a so-called “heel effect” exists. The heel effect is a decreased dose rate on the side of the x-ray tube target.^19^ The heel effect is affected by the anode angle, with a steeper angle decreasing the dose, leading to increased material for attenuation. The heel effect is more prominent with larger focal spots. It is also affected by the distance to the detector or subject, with larger distances reducing the effect, but at the cost of a reduced dose rate. In addition, the filtration inherent in the x-ray tube exit window and any added filtration could further affect the x-ray spectrum and penetrability.^20^ Additional collimation and filtration could improve homogeneity. The collimation would reduce field size and limit the area in which there is a larger dose fall-off; however, the penumbra will exist and must be clearly delineated before experimentation. Beam filtration could further change the beam spectrum, creating a harder beam with greater penetrability, slightly improving homogeneity. These are all factors that will affect the beam spectrum and distribution without any medium present; however, for these experiments, a vast array of configurations is possible using common biology experimental tools, which can include mice, petri dishes, or vials, which can also affect field homogeneity.^21^

Recently, commercial machines with dual x-ray tubes have been introduced to both overcome the problem of the heel effect and double the dose rate. As expected, dosimetry of dual x-ray tube units is more complicated, and rigorous study is necessary to ensure accurate dosimetry in animal and cell irradiation. However, to the best of our knowledge, no comprehensive dosimetry study for a dual x-ray tube unit has been reported. In this study, we assessed the radiation field and dose inhomogeneity for a dual x-ray tube cabinet irradiator. The system allows for both single and dual tube operation. While it is clearly advantageous for both tubes to be used simultaneously for both output and homogeneity, some measurements are repeated for single tubes to assess the baseline for quality assurance purposes and to determine the benefit for field homogeneity. In addition, single tubes may be needed for shielded applications such as mouse flank irradiation, in which only part of the mouse is irradiated and the rest is shielded. The aim of this work was to report a physics-based approach for assessing dose distribution in a dual x-ray source system.

## Methods and Materials

### X-ray equipment and calibration

**Figure 1:**
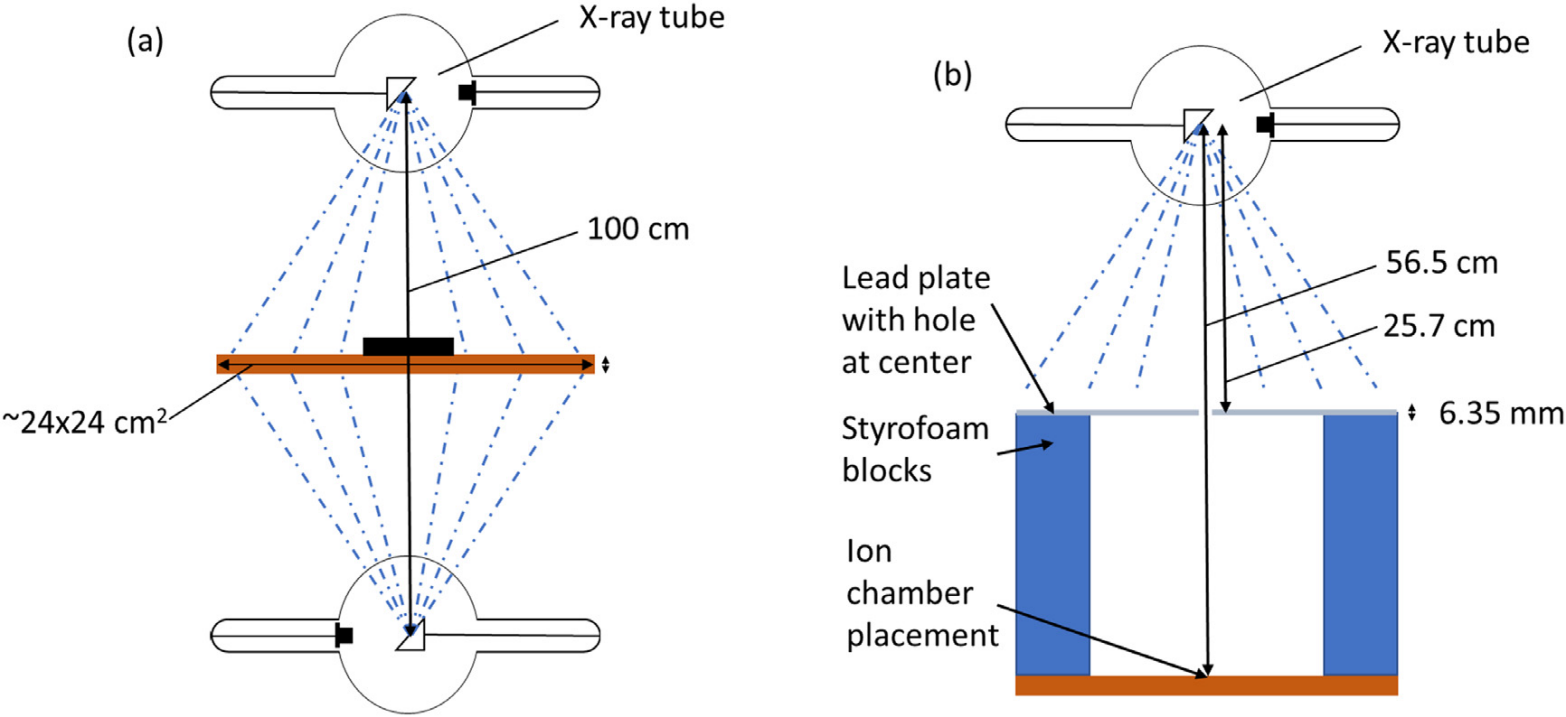
An illustration of the cabinet irradiator configuration for preclinical irradiation (A) and for the HVL measurement (B). The direction of the tubes is opposed to alleviate inhomogeneity due to attenuation of the produced bremsstrahlung photons in the angled anode. The focal spot distance between both tubes is 100 cm. The tray with an approximate area of 24 × 24 cm^2^ can be adjusted plus or minus 6.5 cm. The tray was lowered to 56.5 cm for the HVL measurement. Diagrams are not to scale. *Abbreviations:* HVL = half-value layer.

All irradiations were performed using a CIXD (Xstrahl Limited, United Kingdom) dual head cabinet irradiator. The system is equipped with two x-ray tubes, mounted with focal spots 100 cm apart and cathodes pointed in opposite directions. An illustration of the tube configurations is shown in Fig. 1A. The tubes have a maximum voltage of 225 kVp, a current range of 0 to 13 mA, and a tungsten target with an angle of 30°. The focal spot is 7.5 mm along the largest dimension. The tube has 0.8 mm Be inherent filtration and an additional 1.0 mm Cu filter in place for all measurements. A carbon fiber tray of 0.325 cm thickness is mounted between the tubes and can be adjusted plus or minus 6.5 cm. All measurements were made with the tray at the midpoint between the 2 tubes and the tube set at 220 kVp and 13 mA, unless indicated otherwise. There is no external collimation system, but the specifications note a useful area of irradiation of 18 × 18 cm^2^. As previous studies have reported,^16^^,^^22^ achieving the desired narrow beam geometry for the half-value layer (HVL), as noted in AAPM TG-61,^23^ is challenging, owing to the fixed position between the x-ray tubes. Our measurement was completed with the tray lowered as low as possible for a focal spot distance (FSD) of 56.5 cm (Fig. 1B); however, TG-61 recommends at least a 50 cm distance between the source and detector as well as the attenuating material. We could not achieve a distance of 50 cm between the detector and attenuating material, but that is a common issue with cabinet irradiators, which often cannot achieve that geometry. A lead sheet with a small hole was placed approximately 20 cm above the tray, with the active length of the ion chamber placed within the radiation field as verified by film. Copper plates with thicknesses of 100 µm were placed over the hole in increasing increments until a value beyond the HVL was determined. The actual HVL was interpolated based on the measurements. Only the top tube was measured because both tubes shared the same specifications and parameters and were assumed to have the same HVL.

Absolute output was measured using a calibrated TN30013 ion chamber (PTW Freiburg GmbH, Germany). Measurements were made for both in-air and at-depth protocols, as specified in TG-61.^23^ TG-61 provides 2 formalisms that provide a dose at 2 commonly considered points of interest. The first formalism is the in-air formalism, which is used if the point of interest is at the surface. This does include a backscatter factor that accounts for the phantom material. The second formalism is the in-phantom method, which will determine the dose at the reference depth of 2 cm. There is a chamber-dependent correction factor that is used to account for differences from the in-air formalism. We recommend that readers review TG-61 for an in-depth explanation of both protocols. The full scatter conditions for the in-depth protocol were not achieved, because the recommended phantom is at least 30 × 30 × 30 cm^3^, and the phantom used was 18 × 18 × 4 cm^3^. We also used solid water (GAMMEX) instead of water, based on manufacturer recommendations. It was previously reported that solid water provides an equivalent dosimetry to within 1.5% of actual water for medium energy kilovoltage x-ray units (200-300 kVp).^24^ All appropriate correction factors were applied as per TG-61 protocols, except for the backscatter factor for the in-air protocol. We followed the formalism from Subiel et al, which included an additional correction factor for the lack of full backscatter conditions.^25^ The thickness of the supporting shelf was 3.25 mm, which, based on the formalism and the HVL of our tubes, means that the backscatter factor is considered negligible, and the equation simplifies to the expression for water KERMA, which is equivalent to the dose at the surface for the in-air formalism.

### Relative ion chamber measurements

The relative output was assessed for both tubes simultaneously and for individual tubes. Relative measurements were made with an TN30013 chamber (PTW Freiburg GmbH, Germany). Before measurement, the tray was marked with tape and marker to delineate the distance in both the x direction (along the path of the tube) and the y direction (perpendicular to the path of the tube). The measurements were completed with no build-up cap and for 60 s exposures each.

Measurements were completed along each perpendicular axis and at 4 points along the diagonal axes. We also performed measurements along the central axis for the tray moved up and down 3 cm. The chamber was placed on the tray with no build-up cap for each measurement.

Output variation was assessed over a 2-week period for each tube. The output was measured using solid water placed on top of the tray, with a total thickness of 7 cm. The output was measured using the TN30013 PTW ion chamber. Measurements were repeated twice daily and normalized to the baseline output, which was established immediately following the calibration measurement.

### Irradiation of RadioChromic film

EBT3 GafChromic films (Ashland, Parlin, New Jersey) from a single batch were used for all measurements. Calibration films were irradiated based on exposure time from the corresponding ion chamber measurements for the in-air calibration protocol. We waited at least 20 h before scanning each film. The film was contained first in envelopes and then within a light tight container. An Epson Perfection V800 (Seiko Epson Corporation, Suwa, Japan) was used for film scanning. A calibration curve was generated for the batch of film. The films were scanned in 48-bit mode at 75 dpi in transmission mode, with no image corrections applied. The calibration curve was generated using SNCPatient software (SunNuclear, Melbourne, Florida), and all films were imported into the program. Analysis was completed using an in-house Python script. For each petri dish, a central square region of interest (ROI) was calculated based on the size and resolution of the scanning mode. The central ROI consisted of 50% of the nominal diameter of each petri dish well, unless noted otherwise. The mean dose of each film was reported and compared with the calculated D_w,z = 0_ (the calibrated dose output at the surface of a water phantom as defined by TG-61 using the in-air method) delivered.

#### Film profiles

**Figure 2:**
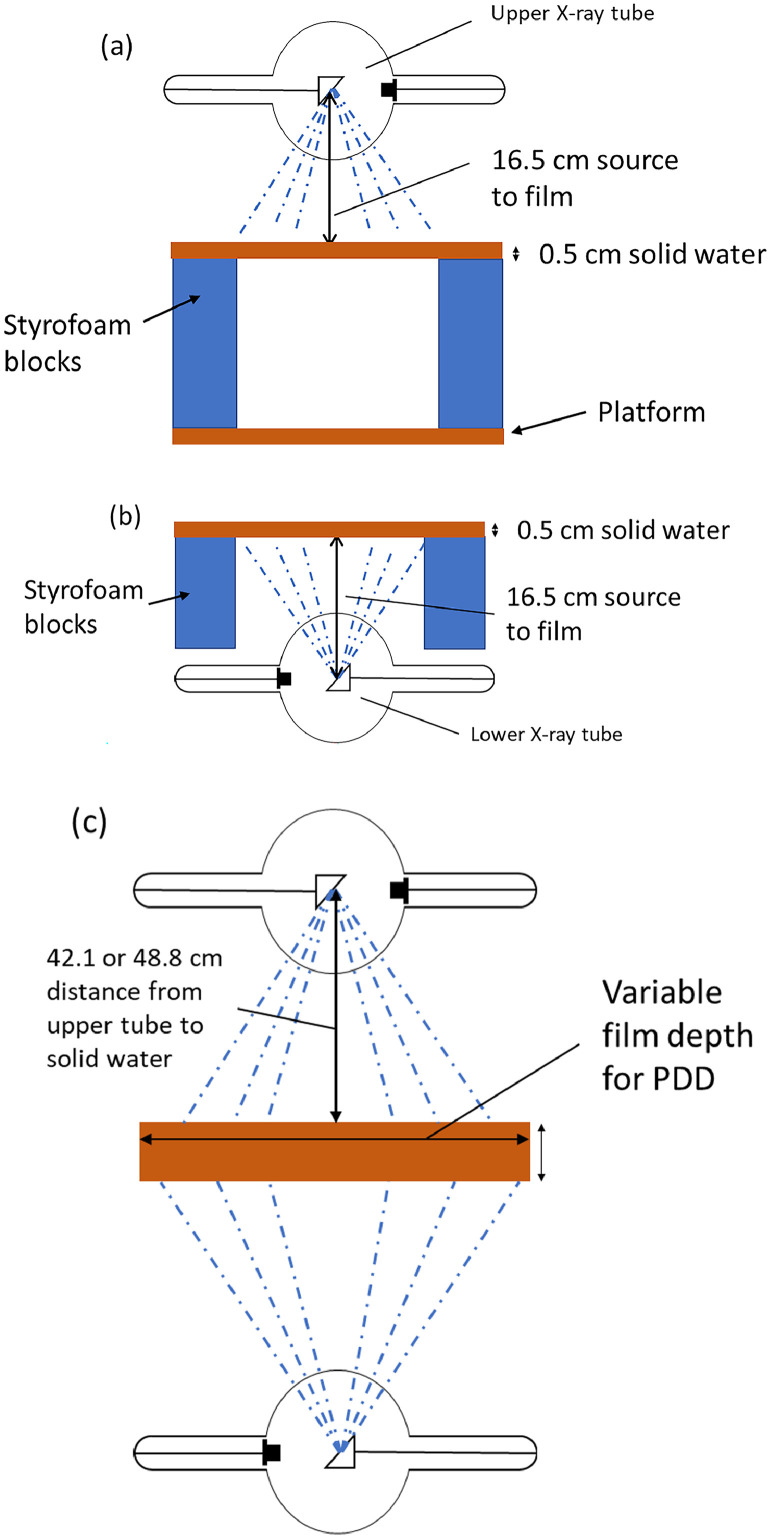
An illustration of the cabinet irradiator configuration for the film profile measurements at 16.5 cm FSD for the top tube (A) and bottom tube (B). An example of the configuration of the PDD_sim_ is shown with the FSD for 2 setups, 42.1 cm and 48.8 cm, and the corresponding solid water thicknesses (C). *Abbreviations:* FSD = focal spot distance; PDD_sim_ = percent depth dose for both tubes simultaneously.

To assess the baseline field homogeneity for each tube, we irradiated film for each tube individually. To capture the whole field for a single tube, a slab of solid water of 0.5 cm thickness was placed on Styrofoam blocks, either on the platform (for the upper tube) or on the floor of the cabinet (for the lower tube) for a focus-to-surface distance (FSD) of 16.5 cm. The diagrams for each setup are shown in Fig. 2A and 2B, respectively.

#### Film percent depth dose (PDD) with solid water

We measured the PDD for each tube individually (PDD_ind_) at 50 cm from the focal spot to solid water (with 5 cm total backscatter) for the lower and upper tubes separately. We then measured the PDD for both tubes simultaneously (PDD_sim_) with 7.9 cm of solid water and 42.1 cm from the focal spot of the upper tube to the solid water. The measurement was repeated with 1.2 cm solid water and 48.8 cm from the focal spot of the upper tube to the solid water. Figure 2C demonstrates the setup for the PDD_sim_ measurements. The PDD_sim_ with 8 cm of solid water was acquired to fully assess the dose deposition at greater depths. While it may not be common to deal with samples or specimens of that thickness, the full PDD_sim_ was acquired to assess the level of dose homogeneity and attenuation at depth compared to those for the single tube irradiators. The tray was left at the midpoint for simplicity, and it was expected that any stacked materials would have a smaller FSD to the upper tube. The system will be shared by various biology research teams who are not familiar with the physics behind the operation of the system, and simple settings are preferred to avoid confusion. The 1.2 cm solid water was estimated to be similar to thicknesses achieved using common petri dish dimensions and liquid volumes.

#### Film measurement with variable solid water and water thickness

We also measured the overall effect of variable solid water thicknesses up to 9 mm with a 3 mm build-up below to determine the range of attenuation and scatter with only a small amount of backscatter to replicate conditions similar to the thickness of petri dishes. The films were placed at a fixed position (same source-to-axis distance), with variable amounts of solid water placed above the film. We also measured the output with variable water volumes under experimental conditions. A single 6 cm diameter petri dish was irradiated with film secured to the bottom using tape on the edge of each film. Water of 4, 6, 12, and 16 mL volumes were placed in the dish. Added saline created an approximate thickness of 1.6, 2.4, 4.7, and 6.3 mm, respectively. The film was taped, so the exact amount of liquid present above versus below the film was not considered, only the total thickness. The thickness of the film was considered negligible. The comparison between the solid water and the petri dishes allowed us to control for any variations due to the geometry and lack of full scatter conditions.

#### Film field homogeneity with petri dish arrangements

To assess the various experimental conditions, we irradiated numerous petri dish apparatuses and a glass slide. We irradiated a large dish (nominal diameter of 15 cm), 7 small dishes (nominal diameter of 6 cm), and a 6-well rectangular apparatus. Each apparatus was filled with 25, 7, and 3 mL, respectively, which corresponds to thicknesses of 6.9, 7.8, 7.5 mm, respectively. The glass slide had 4 small wells that were filled with 1 mL. The experiments using 6 cm petri dishes were repeated with additional dishes placed to assess the effect of various arrangements of dishes on scatter conditions. The large dish was irradiated with 2 stacked to assess output variation between them. All dishes were irradiated based on times calculated using the in-air calibration protocol. The dishes were irradiated for a time that was determined to be equivalent to D_w,z = 0_ = 2 Gy.

#### Film field homogeneity with 3D-printed mouse phantom

**Figure 3:**
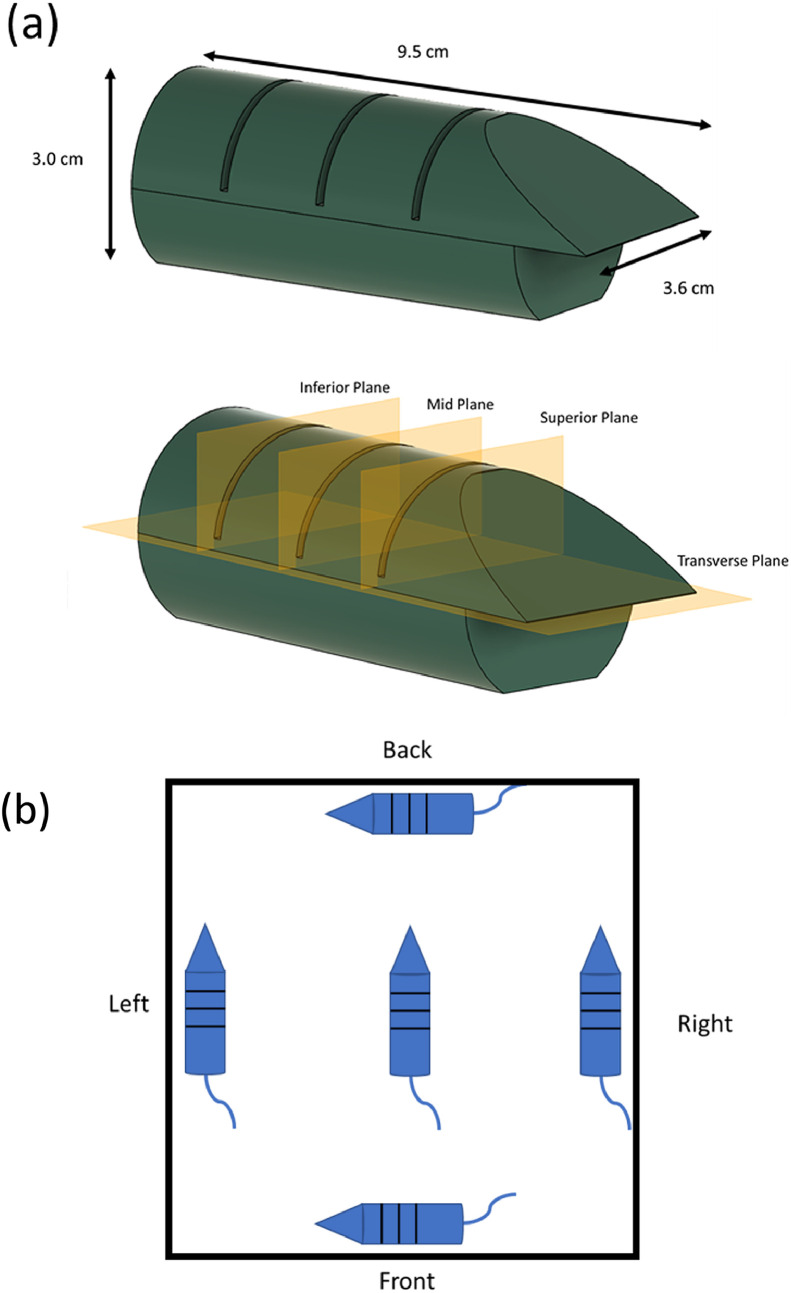
Mouse phantom with experimental setup and film profiles. (A) Mouse phantom modeled in Fusion360. The phantom was cut in half for film placement along its length, with slits along the back (1.85 cm spacing) for additional film to be placed. The phantom was printed with PLA with 100% infill. (B) Diagram of mouse phantom with orientation shown. The phantom was placed at the furthest point along each axis. The black lines indicate the slits for film placement. The diagram is not to scale.

Film was also placed in a 3D-printed mouse phantom with dimensions of 9.5 × 3.6 × 3.0 cm^3^, which was constructed to be roughly cylindrical (Fig. 3A). The mouse was printed in 2 pieces, split along the transverse plane to allow for film to be placed at the midpoint. Three additional slits, labeled superior plane, mid plane, and inferior plane, were printed along the back of the phantom for film to be placed. The spacing between each slit was 1.85 cm. The mouse was printed using only polylactic acid filament (PLA) with 100% fill. The phantom was irradiated 4 times while placed at the farthest points possible on the tray along the x- and y-axes (Fig. 3B). Film was placed between the 2 pieces and within each slit for each irradiation. The pieces of film were cut larger than the phantom, but the extent of the film within the phantom was marked. The films were read out based on the previously mentioned procedure. Only the extent of film that was within the phantom was considered for measurement. Any dose measured outside of the phantom was zeroed out in postprocessing.

## Results

### Output calibration

The HVL was measured as 1.7 mm Cu, with a second HVL of 2.4 mm Cu. The ion chamber was not calibrated for the exact HVL of these tubes, so the calibration factor was interpolated from the reported calibrations. The chamber was calibrated for an HVL of 1.13 mm Cu. The measured absolute output at D_w,z = 0_ was 1.27 Gy/min for both tubes, with the ion chamber placed on the tray at the midpoint (0.65 Gy/min for the upper tube and 0.61 Gy/min for the lower tube). The dose rate as measured here is for the tray centered. The dose rate could be increased for a single tube with a smaller FSD, but at the cost of reduced field size and field homogeneity. The ion chamber was not centered between the 2 tubes exactly at 50 cm FSD owing to the tray, but it was decided that the tray should be left at the midpoint for consistency. The upper tube FSD to the ion chamber central axis was measured as 49 cm. The ion chamber reading was corrected for temperature, pressure, and other factors (P_Q,cham_ and ratio for water-to-air of the mean mass energy-absorption coefficient) outlined in TG-61, except for the backscatter factor, B_w_. However, based on a formalism from Subiel et al for the tray thickness and energy in the CIXD, the backscatter factor effectively becomes zero.^25^ In addition, some minor perturbations to the reference setup were used as recommended by the vendor. Since no collimation mechanism is provided, the recommended 10 × 10 cm^2^ was not used. Therefore, the field size correction to P_Q,cham_ (equal to 1.017) for nonreference field sizes based on the nominal field size of 18 × 18 cm^2^ was applied as specified in TG-61 for the in-phantom measurement. The final perturbation was that GAMMEX solid water was used instead of water for the dose at-depth protocol, as previously mentioned.

### Relative ion chamber measurements

**Figure 4:**
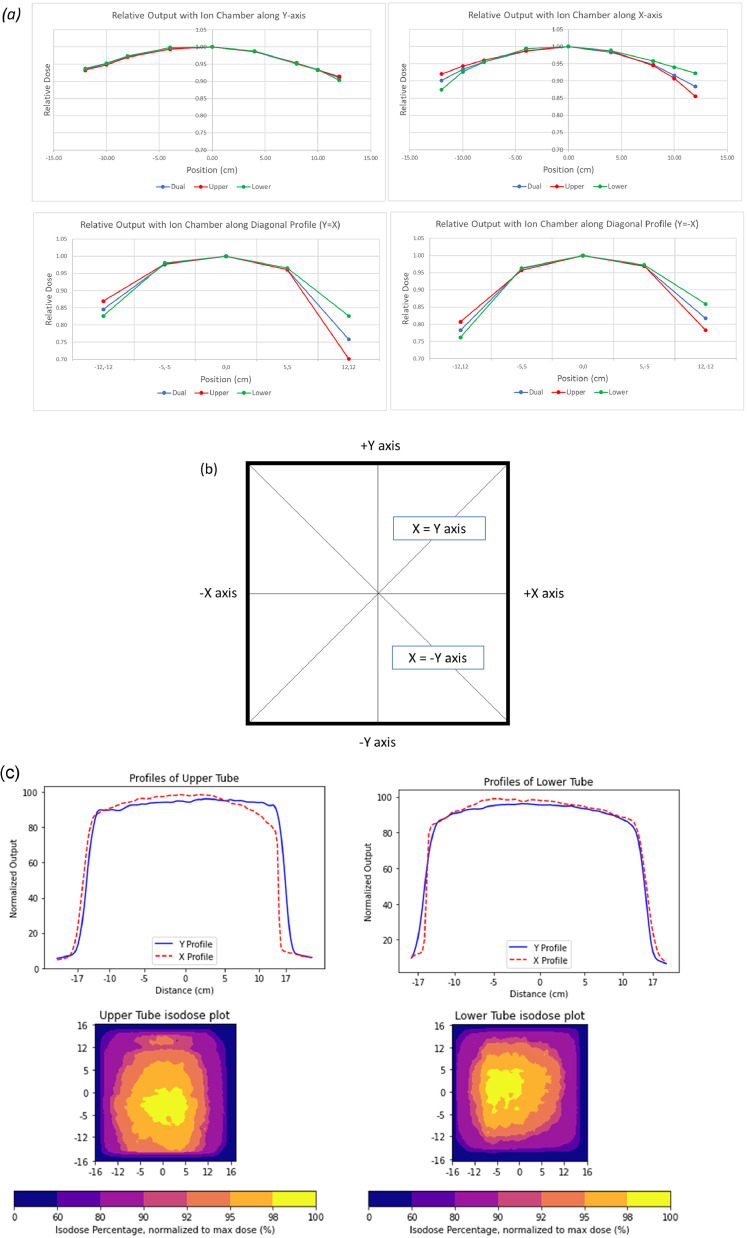
(A) Individual tube and simultaneous tube output along the perpendicular axes, normalized to the output at the central axis for each tube or both tube outputs, and simultaneous tube output along the diagonal axes, normalized to the central axis of each tube or both tube outputs. (B) An outline of the CIXD platform demonstrating the x, y, and diagonal axes. Measurements were acquired along each axis. (C) One-dimensional dose profiles of each individual tube along the x- and y-axis and 2-dimensional dose map of each tube. Dose maps were acquired using EBT3 film measured at a 16.5 cm focal spot distance, scaled to 50 cm here.

Output measurements at various points along both the x- and y-axes and diagonal axes were performed to assess field homogeneity (Fig. 4A). The axes of the tray are shown in Fig. 4B. Each point was measured 3 times, with the average reported. The dose between –10 cm and +10 cm along both axes was within 8.4% of the central axis dose with both tubes on simultaneously. Doses at the corners of the tray decreased significantly at the farthest point, but within the 15 × 15 cm^2^ central area (–7.5 cm to 7.5 cm in both the x and y axis), they remained within 5%. There appeared to be asymmetry along both axes. The output along the y-axis was lower at the +12 cm point by 9%, whereas that at the –12 cm point was lower by 7%. This was also noted along the x-axis, with the +12 cm point lower by 12% and the –12 cm point lower by 10%. However, the reduced dose along the x-axis due to the heel effect was offset by the individual tube output being higher on the cathode side of the target. The dose rate at both 3 cm above and below the midline was within 2% of the measured dose rate at 50 cm. Output variation for both tubes individually and simultaneously were within 1% over the span of 2 weeks.

### RadioChromic film measurements

#### Film profiles

The relative 1D and 2D profiles for the upper and lower tubes are shown in Fig. 4C. The irradiation field appeared largely homogenous, with the heel effect evident along the profiles. The profiles were taken at 16.5 cm FSD to reduce the field size, but the axes were scaled to 50 cm FSD. The field size as measured by the full-width half maximum at 50 cm FSD based on the scaling would be approximately 30 × 30 cm^2^; however, the field within 95% was approximately 15 × 15 cm^2^, which agrees well with the ion chamber measurements.

#### Film PDD with solid water

**Figure 5:**
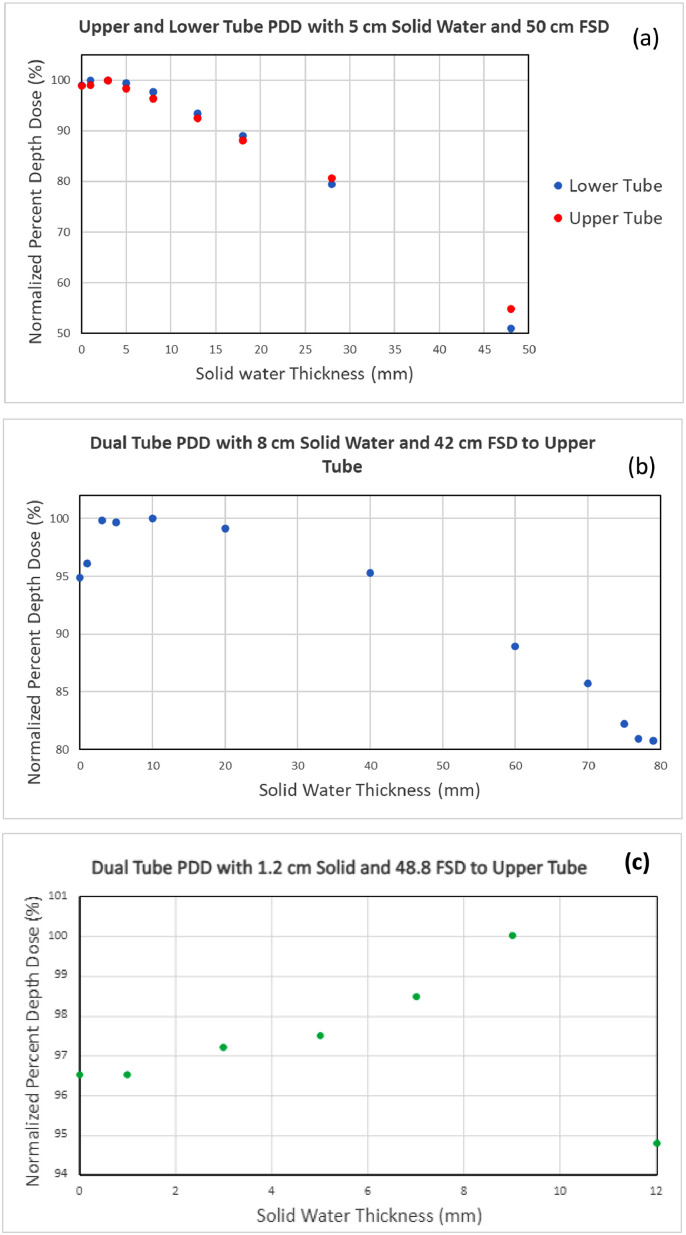
Percent depth dose for single tube exposures with 5 cm of backscatter material and 50 cm FSD to each tube (A) and for simultaneous tube exposures with 7.9 cm backscatter and 42.1 cm FSD to the upper tube (B) and 1.2 cm backscatter and 48.8 cm FSD to the upper tube (C). Each plot has different scale, owing to differences in FSD and backscatter thickness. *Abbreviations:* FSD = focal spot distance.

The PDD_ind_ (50 cm FSD to each tube) and PDD_sim_ (42.1 cm FSD to top tube focal spot) are shown in Fig. 5A and 5B, respectively. Figure 5A demonstrates good agreement between both tubes individually, with a slight difference at the most distal position. The cause is unclear, but there are slight differences in the beam path between the upper and lower tubes due to the manufacturing of the tray. In Fig. 5B, the PDD_sim_ with 7.9 cm solid water shows that the output is within 95% of the maximum dose for 4 cm (ie, from the depth of zero to the depth of 40 mm in the figure) with the additional scatter conditions. The additional solid water depth of 7.9 cm is not relevant for cell cultures or mice. Rather, it is an extreme scenario that does demonstrate a lower dose decrease at depth than that for the individual tubes. Figure 5C represents a more likely scenario in which the thickness is similar to what may be expected for a petri dish. The PDD_sim_ with a 1.2 cm total thickness, as shown in Fig. 5C, has the maximum dose closer to the top tube. The difference is minor, with all film measurements within 6% of the maximum dose.

#### Film measurement with variable solid water and water thickness

The variable solid water thickness demonstrated a build-up region, in which the dose at surface thickness is about 8% lower than the maximum mean dose at 7 mm. The variable water thickness in the petri dish showed minimal variation over the small thicknesses. A slight increase was seen with increasing solution; however, the variable ROI mean doses were within 7% of the measured maximum mean dose at 6.3 mm depth. This agrees well with the solid water measurements. The results for both the solid water and petri dish with variable medium are shown in Table 1.

**Table 1:** Mean dose of the film region of interest normalized to the dose maximum for variable thickness measurements

Solid water*		Petri dish with water†	
Thickness (mm)	Normalized mean	Thickness (mm)	Normalized mean
0	0.92	-	-
2	0.94	1.6	0.93
3	0.96	2.4	0.97
5	0.99	4.7	0.99
7	1.00	6.3	1.00
9	0.99	-	-

⁎ Solid water measurements were made with variable thicknesses up to 9 mm, with a 3 mm backscatter.

† The petri dish measurements were made in a 6 cm dish with variable water volumes up to 6.3 mm, with their corresponding thicknesses shown here.

#### Film field homogeneity with petri dish arrangements

**Figure 6:**
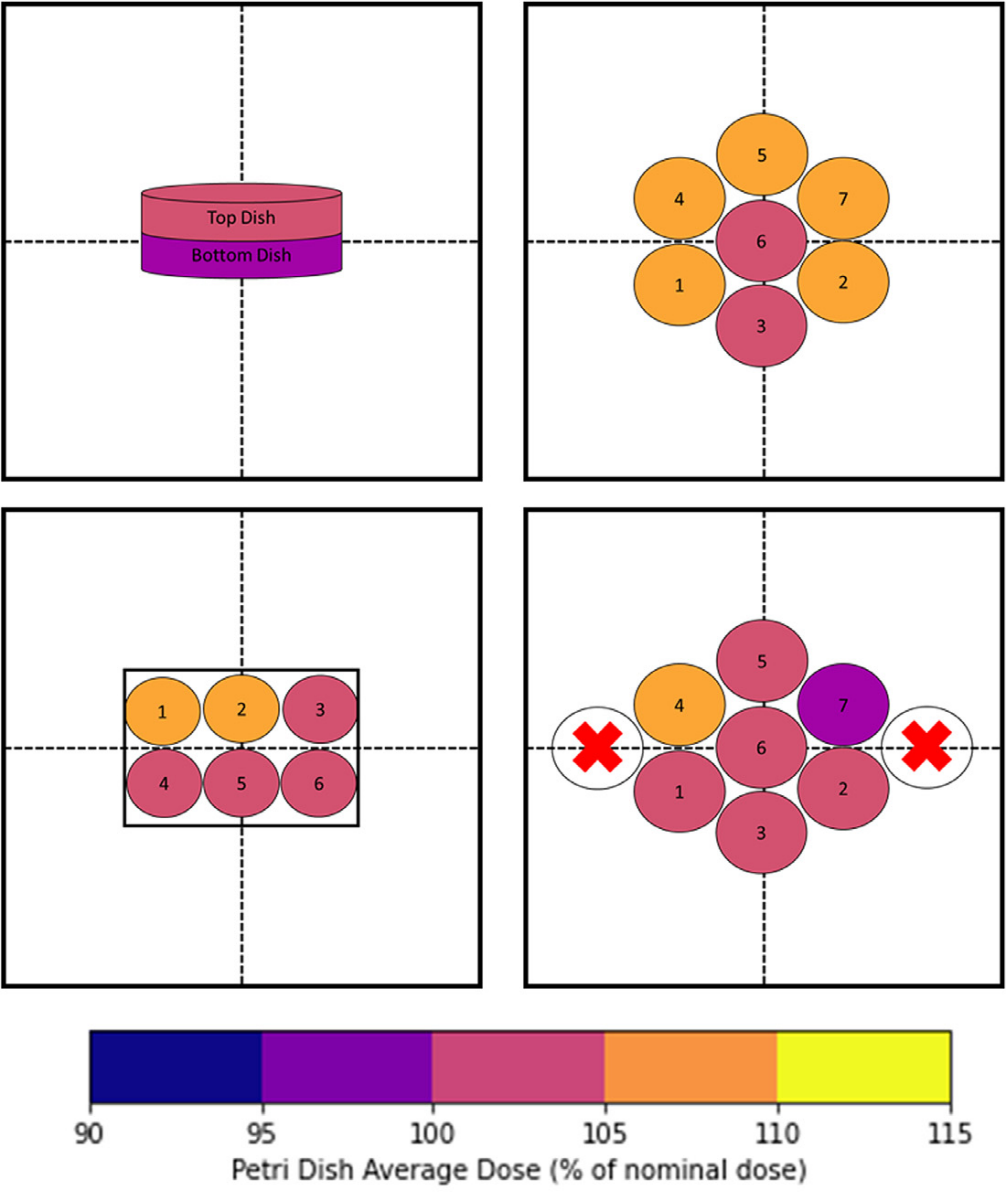
Arrangement of various petri dishes on the available 24 × 24 cm^2^ mounting tray with the dose measured with EBT3 film relative to the nominal dose as calculated with D_w,z = 0_. The dishes and tray are not to scale, and the 15 cm dishes are shown in 3D for reference, with the other arrangements shown in 2D. The 2 stacked 15 cm petri dishes centered on the mounting tray (top left). The 6 cm petri dishes in the first arrangement (top right). The 6 cm petri dishes in the second arrangement, with no dose measured at the furthest point on the left and right (bottom right). The 6-well petri dish centered on the tray (bottom left).

The numerous petri dish film exposures, based on the in-air calibrated doses, yielded an overall homogenous dose for each configuration. All film measurements were within 7% of the prescribed dose based on the in-phantom calibration, which we described as the nominal dose. An illustration of each dish arrangement and the relative mean dose to the ROIs is shown in Fig. 6. There were minor deviations between the 6 cm dishes that were tested with 2 configurations. In the first iteration, with only 7 dishes, all dishes received a mean dose to the central ROI of at least 100% of the nominal delivered dose, with a range of 101.7% to 106.2%. When the experiment was repeated with additional dishes placed, no discernable difference was noted; however, 1 dish did receive lower than the nominal dose, but only by 3%. The petri dish with 6 wells had good agreement between the nominal dose and the dose in each well. The mean dose to the central ROI ranged from 101.3% to 106% of the nominal dose. The 15 cm stacked dishes had central axis mean doses within 2% of each other and the nominal dose, which is very close in agreement and would point to this configuration being an efficient form of delivery. However, further work would be needed to assess how many dishes could be stacked while maintaining homogenous delivery. The mean dose for each well in the 4-well glass slide was within 5% of the nominal dose.

#### Film field homogeneity with 3D-printed mouse phantom

**Figure 7:**
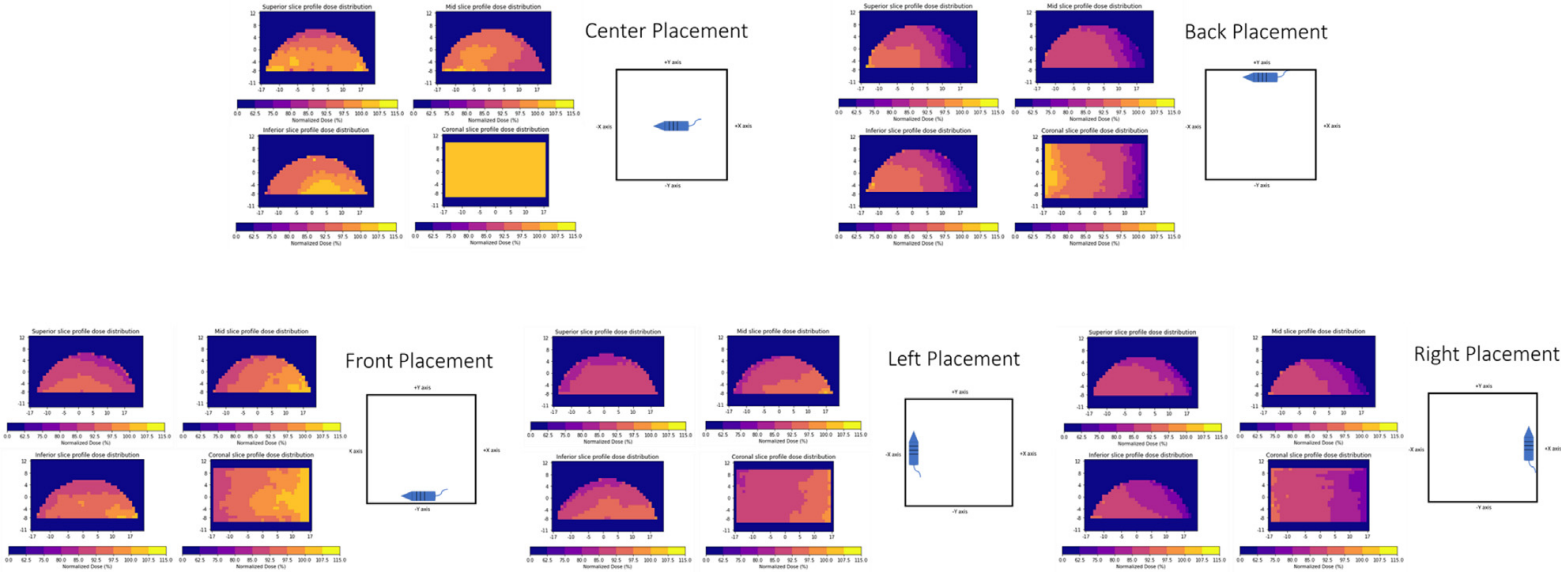
Film profiles for each slit along the top portion of the mouse phantom and film profile within the coronal slice of the phantom with the normalized dose displayed (normalized to 200 cGy). The irradiated film outside the extent of the phantom is zeroed out for display purposes.

The 3D-printed mouse phantom profiles for each plane with the phantom at the different axes are shown in Fig. 7. The asymmetry of the profiles is in line with the ion chamber measurements, with most of the profiles receiving at least 85% of the nominal dose. The lowest dose was received by the phantom placed at the +x-axis, where it received 70% of the nominal dose. These profiles represent a “worst-case scenario” in which the mice would be placed at the periphery of the field, and the phantom provides a mechanism for testing potential placement for an estimation of the expected dose.

## Discussion

Recent publications have espoused the need for radiation physicists to not only be involved in the commissioning of new x-ray cabinets, which are taking the place of Cs-137 irradiators, but also ensure accurate dose parameters for any new setup. X-ray cabinet irradiators are well suited for various types of sample irradiation, but as well documented in other recent papers,^15^^,^^16^^,^^25^^,^^26^ the given calibrations from TG-61 protocols rely on conditions that are generally not present in experimental irradiations. While the goal of this study was not to experimentally validate previous findings, we did find that the individual tube measurements for the PDD agreed well with the previously tabulated data, matching within 5% across the measured depths for their reported 2 mm Cu HVL PDD with a 20 cm field and 5 cm backscatter.^25^ It can be challenging to compare across x-ray units owing to variations in energy spectrum, filtration, and geometry. Even the specified matching PDD does not agree with our units’ parameters, which may account for the variation. However, the benefit of this system is the dual head design, which allows for increased dose rates and better homogeneity at depths. For a single tube with a backscatter of 9 mm, the dose at 9 mm depth would be 18% less than that at the surface.^25^ We found that the dose with the dual tube design remained within 92% of the maximum dose with 12 mm of backscatter (Fig. 5C) and was within 6% of the maximum dose for a variable saline solution with a maximum depth of 4.7 mm (Fig. 6).

The other potential benefit of the dual tube design is the reduction of the heel effect, with the direction of the tubes opposed. The benefit is evident in the profile as measured by the ion chamber, with a single tube on or with both tubes on simultaneously. The tray that holds samples, dishes, and animals is approximately 24 × 24 cm^2^; however, the dose falls off by approximately 10% at the top part of the tray (+y-axis), which is in line with other single tube devices.^20^ As the heel effect is primarily a function along the anode-cathode direction, the dual tube design improves the dose fall-off along the x direction, but only minimally along the y direction.

As noted in numerous publications, not only is there variability in the calibration and irradiation conditions of many preclinical irradiators, but also a wide possibility of experimental conditions.^15^^,^^16^^,^^21^ Preclinical irradiators can be used to irradiate cells in petri dishes, test tubes, whole body mice, and partial body mice. The CIXD does not have any corresponding imaging capabilities, so even simple dose computational methods that are available in other preclinical devices such as the small animal radiation research platform or SAARP (Xstrahl) are not available. Previously published data, such as the British Journal of Radiology Supplement 25 (BJR-25)^27^ has been a potential guide for comparison, but generally, it assumes infinite backscatter. Recent publications have remedied these issues but cannot fully capture the highly variable conditions resulting from a myriad of irradiators and experimental conditions.^14^^,^^15^^,^^16^^,^^28^

Therefore, we are reliant upon using either physics-based dosimetric tools or biologic-based measurements. There are various dosimetric tools that are commonly used in clinical machines that operate at the MV energy range (these include ion chambers, metal-oxide-semiconductor transistors, thermoluminescent dosimeters, and radiochromic film); however, as noted in other publications, there are some limitations for certain dosimeters.^11^^,^^21^ While the calibration should always be done with an ADCL calibrated ion chamber, it is much more difficult to measure the dose under experimental conditions. In an initial study outlining the importance of the dosimetry protocol for cell irradiation, Dos Santos et al used a parallel plate chamber and film to measure the dose for petri dishes that were irradiated with the SAARP, with both the film and parallel plate chamber set below the petri dish.^16^ However, this setup is not possible for our dual tube geometry, and the film is not placed exactly at the point of interest, which is within the petri dish. In an additional paper, Dos Santos et al recommend using the film placed within the container filled with medium, which is in line with our methodology.^28^

The thickness measurements with both the petri dishes (variable solution) and the solid water agree well (Table 1). Both the petri dish and solid water measurements were all within 8% of the maximum dose delivered using the minimal thicknesses. As expected, based on the dual tube PDDs, both the variable thickness petri dish and the solid water measurements were within the build-up region, showing an increase based on thickness. This is generally not seen in conventional single tube low-to-mid kV irradiators, in which there is rapid fall-off at depth. The stacked 15 cm petri dishes showed a negligible dose difference between the upper and lower dishes, which further demonstrates the homogeneity at various depths (Fig. 6). All except 1 petri dish in the experimental conditions received at least the nominal dose, with the largest deviation being 6.2%. Based on the volumes in each dish or well, the thickness of material was in the range of 7 to 8 mm when combined with the thickness of the petri dish base and lid. Compared with the PDD_sim_ with 1.2 cm solid water backscatter, there was a build-up region of approximately 0 to 10 mm, but the dose at the surface was only 3.5% lower (Fig. 5C). A previous study showed a doubling effect of irradiation with 120 kVp x-rays in γH2AX foci per gray;^29^ however, we noted that for our 220 kVp tube, there was a minimal increase in dose on the glass slides as measured by film. Based on both the solid water and petri dish measurements, the system was relatively insensitive to deviations in the thickness of the medium used with commercial petri dishes, but depending on the accuracy required of radiobiological experiments, strict procedures may need to be implemented.

Our relative measurements with our mouse phantom were in good agreement with the ion chamber measurements along the perpendicular axes. While the mouse phantom, made of PLA (Z_eff_ = 7.25), has a Z_eff_ close to that of water and soft tissue, it cannot take bone effect into account. However, bone effect in mice is considered not significant. For instance, Esplen et al created a more realistic mouse phantom but found that the dose measured without a bone-mimicking material only reduced the dose to the brain by 2%.^30^ They also noted that their phantom, made of 3D printing materials meant to mimic polymethyl methacrylate, actually resulted in a dose underestimated by 15%, owing to differences in the mass-energy absorption coefficient at the mean energy of the SAARP. Based on a Z_eff_ similar to that of water, the PLA mouse phantom should more accurately mimic the soft tissue of a mouse. Each film within the mouse phantom exhibited some level of dose gradient (Fig. 7). Even the phantom placed at the central axis had a dose of around 92% of the nominal dose. This was primarily at the edge of the film, where a dose gradient is to be expected. Compared with the mouse placed at the central axis, the mice at other placements showed that placement greatly affected the dose distribution, with the lowest dose being 70% of the central axis dose. This can be a cause for concern if the mice are not properly restrained and not limited to staying within the 15 × 15 cm^2^ area, which is expected to be within 5% of the central axis dose, as shown by the ion chamber measurement.

The mouse phantom will be used to perform additional testing as required to ensure that mouse irradiation is within expected dose levels and can be used for future studies that perform partial irradiation, such as studies of flank tumors. It is important to note that mouse irradiation can be complicated by many additional factors including mouse strain, timing of irradiation, diet, anesthesia, and of course, radiation variables.^31^ While our 3D-printed model may be appropriate for certain estimations, a more robust or target model may be needed for specific applications such as whole thorax lung irradiation. We recommend that the dose should be approximated with phantoms and film before irradiation to determine the overall uncertainty for a certain setup geometry and immobilization. Further targeted irradiations may require the use of an image-guided radiation therapy machine, such as the SAARP.

Uncertainty has been previously discussed regarding measurements surrounding output calibration with ion chambers and film for both MV and KV units.^16^^,^^32^ The largest uncertainty regarding the output calibration is the beam quality difference between the calibration energy and our devices’ energy.^23^ It was conservatively estimated that the total uncertainty was from 3% to 5%, assuming variables such as correction factors, the warm-up effect, and statistical variability in measurements. TG-61 notes that in addition to the difference in beam quality, the greatest sources of uncertainty are the various correction factors used (backscatter factor, P_Q,cham_, and water-to-air ratio of the mean mass energy absorption coefficient).^23^ For film, the ion chamber calibration contributes to the uncertainty of the film calibration. Other factors such as scanner warm-up time, varying sensitivity in the scanner bed, and film optical density measurement will also contribute to the uncertainty. For film calibration, it has been previously estimated that there is an uncertainty of 4% (k = 2).^16^

This study was limited by a lack of additional biologic benchmarking with established cell lines and mouse studies to further validate and establish the biologic endpoints for certain known dose levels. In addition, no statistical tests were used as part of this study. As this work was part of the CIXD commissioning, we hope to further establish these endpoints with additional studies and compare our results to those of previous studies with our cesium irradiator. We primarily focused on physics-based dosimetric tools for validation, because that should always be the first step for preclinical devices.

The recommendations by Bucher et al and various other studies are echoed here.^11^^,^^15^^,^^16^^,^^21^^,^^28^ Caution should be used when relying upon machine specifications, such as the useful area of irradiation, as this information does not account for experimental conditions, and what is considered useful may vary significantly depending on the experiment's required level of accuracy. It is recommended that the sample vessels use a low absorbent material, with a minimal amount of liquid (more on this below), and standardized positions or holders to allow reproducible positioning. The dual tube design of the CIXD dual head irradiator allows for the majority of the tray to be used. In addition, the dual tube widens the homogeneity at depth, with the doses in the phantom all within 7% of the maximum dose at variable liquid thicknesses up to 0.63 cm with no additional backscatter. Since there is no drastic attenuation that is commonly seen in single x-ray tube designs, various amounts of medium can be used without compromising the dose at depth, even based on the dose rate at the surface using the equipment outlined in this paper. Regardless, the dose should be well characterized for each unique experimental condition, and the dose as determined by ADCL calibration cannot be used without proper experimental assessment. This will require collaboration between radiation physicists, radiation biologists, and vendors in the determination of proper dose specifications.

## Conclusion

X-ray irradiators are becoming standard for preclinical experiments, and while some models have been well assessed, many assumptions and previous dose tabulations become less accurate under certain experimental conditions. This work used previously established guidelines for the measurement of a dual head irradiator and demonstrated that accurate dosimetry is required for each unique experimental condition. To ensure that dosimetry is performed accurately with all radiologic variables accounted for, a medical physicist should be consulted during commissioning of irradiators and during study design.

## Disclosures

The authors declare that they have no known competing financial interests or personal relationships that could have appeared to influence the work reported in this paper.
